# Decoding the intensity of sensory input by two glutamate receptors in one *C. elegans* interneuron

**DOI:** 10.1038/s41467-018-06819-5

**Published:** 2018-10-17

**Authors:** Wenjuan Zou, Jiajun Fu, Haining Zhang, Kang Du, Wenming Huang, Junwei Yu, Shitian Li, Yuedan Fan, Howard A. Baylis, Shangbang Gao, Rui Xiao, Wei Ji, Lijun Kang, Tao Xu

**Affiliations:** 1 National Laboratory of Biomacromolecules, CAS Center for Excellence in Biomacromolecules, Institute of Biophysics, Chinese Academy of Sciences, Beijing, 100101 China; 2 Key Laboratory of Molecular Biophysics of the Ministry of Education, College of Life Science and Technology, Huazhong University of Science and Technology, Wuhan, 430074 China; 3 Institute of Neuroscience and Department of Neurosurgery of the First Affiliated Hospital, Department of Neurobiology, NHC and CAMS Key Laboratory of Medical Neurobiology, Zhejiang University School of Medicine, Hangzhou, 310058 China; 4 College of Life Sciences, University of Chinese Academy of Sciences, Beijing, 100049 China; 5 Department of Zoology, University of Cambridge, Cambridge, CB2 3EJ UK; 6 Department of Aging and Geriatric Research, Institute of Aging, College of Medicine, Center for Smell and Taste, University of Florida, Gainesville, 32610 FL USA; 7 Center for Biological Instrument Development, Core Facility for Protein Research, Institute of Biophysics, Chinese Academy of Sciences, Beijing, 100101 China

## Abstract

How neurons are capable of decoding stimulus intensity and translate this information into complex behavioral outputs is poorly defined. Here, we demonstrate that the *C. elegans* interneuron AIB regulates two types of behaviors: reversal initiation and feeding suppression in response to different concentrations of quinine. Low concentrations of quinine are decoded in AIB by a low-threshold, fast-inactivation glutamate receptor GLR-1 and translated into reversal initiation. In contrast, high concentrations of quinine are decoded by a high-threshold, slow-inactivation glutamate receptor GLR-5 in AIB. After activation, GLR-5 evokes sustained Ca^2+^ release from the inositol 1,4,5-trisphosphate (IP_3_)-sensitive Ca^2+^ stores and triggers neuropeptide secretion, which in turn activates the downstream neuron RIM and inhibits feeding. Our results reveal that distinct signal patterns in a single interneuron AIB can encode differential behavioral outputs depending on the stimulus intensity, thus highlighting the importance of functional mapping of information propagation at the single-neuron level during connectome construction.

## Abstract (web summary)

Little is known about how stimuli of different intensities result in different behavioral outcomes in *C. elegans*. In this study, the authors demonstrate how distinct signal patterns, involving different glutamate receptors, in a single interneuron AIB can encode differential behavioral outputs depending on the stimulus intensity

## Introduction

Decoding stimulus intensity is a fundamental property of sensory systems. Organisms use this information to adapt their behavior in response to stimulus strength. For instance, in many vertebrates and invertebrates salt can be either attractive or repulsive depending on its concentration^1–3^. This leads to important questions in neuroscience, how is the stimulus intensity encoded at different stages of neural processing and how is this information translated into complex behavioral outputs?

Simple organisms such as *C. elegans* provide premier platforms to tease out how signaling molecules and neuronal circuits generate complex behaviors. *C. elegans* is equipped with a small nervous system, merely 302 neurons and ~7000 synapses, of which the complete connectome is known^4^. Moreover, *C. elegans* responds to complex environmental cues associated with the senses of smell, taste, touch, and temperature, all of which evoke multifaceted behavioral responses such as locomotion, feeding, dauer formation, social behavior, and also learning and memory^5–9^. Like many vertebrates and invertebrates, *C. elegans* is capable of decoding stimulus intensity and translating it into multifaceted behavioral outputs. For example, anterior gentle touch typically triggers short reversals followed by forward movement without a direction change. By contrast, anterior harsh touch evokes long reversals, which are often followed by a directional change through omega turns^10^.

Previously we and others have shown that quinine, a type of bitter alkaloid and repellent to *C. elegans*, can generate two types of behavior: reversal initiation and feeding suppression^11,12^. This phenomenon provokes some intriguing questions. First, do reversal initiation and feeding suppression always occur simultaneously when encountering quinine or do they occur differentially depending on the stimulus intensity? Additionally, what are the underlying neural circuitry and molecular mechanisms for signal decoding, processing, and different type of behavioral output?

Several key components in the neural circuit that control *C. elegans* reversal initiation have been previously identified^13–17^. In particular, ASH is the main sensory neuron responsible for the detection of repellents detection including quinine while ASK plays a minor role^11,18^. Moreover, a group of command interneurons (AVA, AVD, and AVE) play an important role in the initiation of reversals. The ablation of AVA and AVD severely reduces the rate of spontaneous reversals but does not abolish them entirely^15,19^. ASH sends synapses to both AVA and AIB^4^. Glutamate released from the ASH sensory neuron is necessary to activate both AIB and AVA in reversal initiation^15,16^. Mutants in a glutamate receptor *glr-1* are significantly defective in the reversal response^15–17^. Targeted expression of GLR-1 to the interneurons AVA or AIB results in a partial rescue of the reversal response, supporting a model in which the AIB and AVA pathways function in parallel, but not completely redundantly^15^. It has also been suggested that RIM acts downstream of AIB and is involved in reversal behavior^13,15^. However, RIM also sends chemical and electrical synapses to AIB and generates variability in odor-induced reversals^20^.

One of the most prominent behaviors in animals is feeding. In contrast to locomotion, the circuitry of feeding regulation in *C. elegans* has not been extensively characterized. Previously we have identified a central flip-flop circuit that integrates two contradictory sensory inputs in regulating feeding behavior^12^. The central integration circuit receives inputs from different sensory modalities in such a way that neurons sensing attractive inputs (AWA and AWC neurons) are linked to NSM neurons to facilitate pharyngeal pumping and neurons sensing repellents (ASH neuron) transmit signals to RIM/RIC neurons to suppress pumping. Sensation of quinine suppresses feeding through ASH–RIM pathway. Therefore, it appears that the signals encoding the reversal initiation and feeding suppression converge on RIM. The question remains as to how the same sensory signal is decoded and transmitted to the same interneurons to control two types of behavioral outputs.

By integrating Ca^2+^ imaging, optogenetics, genetic manipulation, and electrophysiology at the single-neuron resolution, here we reveal that a single-interneuron type, AIB, decodes the intensity of quinine stimuli and encodes two types of behavioral outputs, reversal initiation and feeding suppression. In AIB neuron, low-concentration quinine input is decoded by a low-activation threshold, fast-inactivation glutamate receptor GLR-1 to mediate the reversal response, whereas high-concentration quinine stimulation is decoded by a high-activation threshold, slow-inactivation glutamate receptor GLR-5 to mediate feeding suppression. These two behavioral outputs are encoded in AIB by distinct Ca^2+^ response patterns: small, transient [Ca^2+^]_i_ spikes for short reversals, and large, sustained Ca^2+^-release from IP_3_-sensitive Ca^2+^ store for long reversals and feeding suppression. Together, our data reveal the circuit, synaptic, molecular, and intracellular mechanisms by which *C. elegans* decodes the intensity of quinine stimulus and encodes two behavioral outputs.

## Results

### Simultaneous Ca^2+^ imaging and behavioral tracking

To further dissect the circuit connections and molecular mechanisms that regulate both feeding and locomotion, we developed a fast tracking system called iCaN (imaging the Calcium activity of Nematodes) that not only analyzes locomotion and feeding behavior, but also simultaneously monitors intracellular Ca^2+^ concentrations ([Ca^2+^]_i_) in the neurons of freely moving worms. Briefly, iCaN is equipped with a dual objective system: (1) a low-magnification objective above the sample to track the movement of free-moving worms and to record feeding behavior (quantified by pharyngeal pumping rate); (2) a high-magnification objective below the sample to monitor neuronal [Ca^2+^]_i_ activity (Supplementary Figure 1). The recorded locomotion is fed back to an x–y stage to center the worm in the field-of-view of both objectives. This design ensures fast 4D imaging of worm neurons at high resolution.

**Fig. 1:**
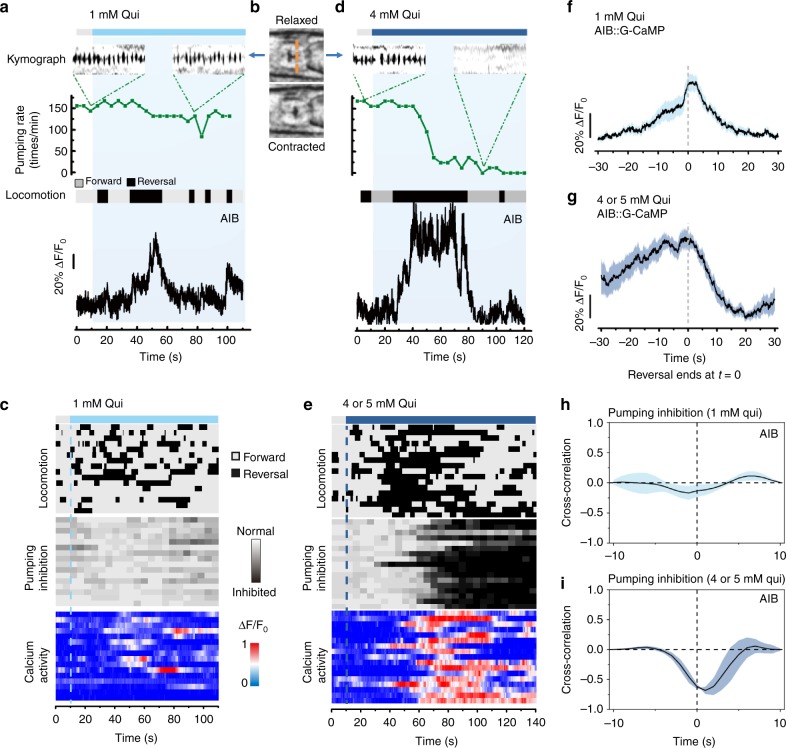
Different strengths of quinine elicit different behavioral outputs: reversal initiation and feeding inhibition. **a**, **b**, **d** 1 mM quinine initiated frequent reversal, induced small and transient [Ca^2+^]_i_ activities in AIB, but did not significantly suppress pumping rate. In contrast, 5 mM quinine-induced pumping suppression, reversal initiation and large, sustained [Ca^2+^]_i_ elevations in AIB. Pumping rate and locomotion were assayed with the iCaN system in a freely behaving worm, while intracellular calcium transients from AIB were simultaneously imaged. The kymographs denote the pumping pulse of feeding in 5 s selected before and after the application of quinine. The grinder muscle moving from relaxed position to contracted position (**b**) and returning to relaxed position represents a pump, which is shown as a spike in the kymographs (**a**, **d**). Orange dash line (**b**) indicates the position selected for the kymographs. The ethograms describing the locomotion behavior were generated automatically from the worm’s posture and the direction of locomotion acquired by the iCaN system. **c**, **e** Locomotion, pumping behavior, and AIB calcium dynamics in response to low (**c**) and high (**e**) concentration of quinine in freely behaving worms. Traces are ordered according to the time of quinine application (dash lines). *n* ≥ 16 worms. **f**, **g** AIB calcium level exhibited an increase during reversals and a decrease at the end of reversals induced by both low (**f**) and high concentration (**g**) of quinine. *n* = 9 worms for high concentration and *n* = 12 worms for low concentration. **h**, **i** Cross-correlation analysis exhibited that AIB calcium activity correlates with pumping inhibition at high concentrations of quinine condition but not at low concentration. Shading around traces indicates error bars (s.e.m.). *n* ≥ 15 worms

Employing the iCaN system, we confirmed that repellents, such as quinine, could generate two kinds of behavioral outputs, reversals in locomotion and suppression of feeding. Interestingly, the two behavioral outputs were not always induced simultaneously but rather depended on the concentration of quinine. Low concentrations (1 mM) initiated frequent reversals but did not significantly suppress pumping rate (Fig. 1a–c), whereas high concentrations (4–5 mM) induced long-lasting reversals and pumping suppression (Fig. 1d, e).

In *C. elegans*, ASH is the main sensory neuron responsible for quinine detection and feeding inhibition^11,12^. AIB is the first layer interneuron downstream of ASH that is involved in reversal initiation^4,15,16^. Simultaneous monitoring of behavior and [Ca^2+^]_i_ in the AIB neurons revealed that low concentrations of quinine-induced small, transient [Ca^2+^]_i_ increases (Fig. 1a, c), while high concentrations of quinine-induced large, sustained [Ca^2+^]_i_ elevations (Fig. 1d, e). We found a good correlation between AIB [Ca^2+^]_i_ activities and reversal behavior. Upon weak stimulation (1 mM quinine), AIB [Ca^2+^]_i_ transients peaked at the end of a reversal and gradually returned to baseline with the resumption of forward movement (Fig. 1f), which are consistent with previous studies^15,21^. In the presence of higher concentrations of quinine, AIB [Ca^2+^]_i_ activity was sustained throughout the long-lasting reversal and began to decline at the resumption of forward movement (Fig. 1g). On the other hand, while the low concentrations of quinine-induced transient AIB activities showed little correlation with pumping suppression (Fig. 1h), the high concentrations of quinine-triggered strong, sustained AIB activities correlated well with pumping suppression (Fig. 1i). Collectively, these results confirm that AIB is involved in locomotion reversal^15,21^ and, more importantly, suggested a previously unknown function of AIB in regulating feeding behavior.

### AIB regulates reversal initiation and feeding suppression

**Fig. 2:**
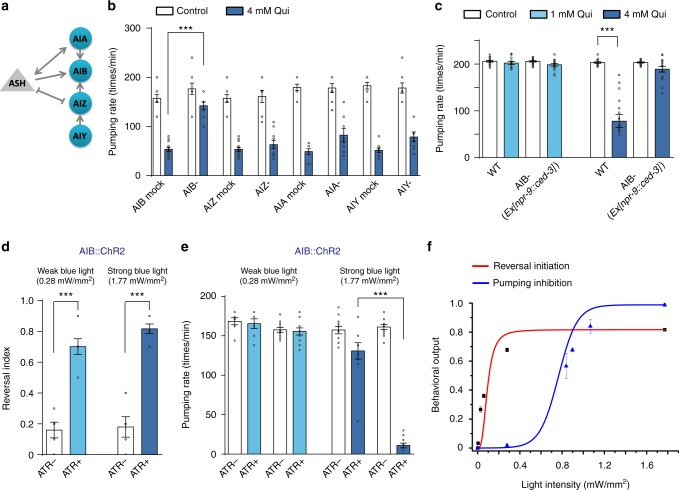
AIB is required for both reversal initiation and pumping inhibition. **a** Schematic showing the connectivity of ASH and the first layer interneurons (AIA, AIB, AIZ, and AIY). **b**, **c** Laser ablation (**b**) and chemical deletion (**c**) of AIB exhibited a significantly defect in high-concentration quinine-induced pumping inhibition. For chemical deletion of AIB, CED-3 was transgenetically expressed in AIB driven by the *npr-9* promoter. Error bars: s.e.m.. Laser ablation: *n* ≥ 8 worms; Chemical deletion: *n* = 16 worms. ***p < 0.001 (*t* test). **d**, **e** Optogenetic stimulation of AIB evoked by either low power (0.28 mW mm^−2^) or high power (1.77 mW mm^−2^) blue light (470 nm; 30 s pulse) triggered reversals, but only by high power blue light-induced pumping inhibition. Worms expressing ChR2 specifically in AIB under the *npr-9* promoter were tested. The transgenic animals cultured on ATR-free plates were taken as control. **d** Reversal behavior. **e** Pumping behavior. Error bars: s.e.m.. Reversal index, *n* ≥ 5 groups, and 10 worms/group; Pumping rate, *n* ≥ 10 worms. ***p < 0.001 (*t*- test). **f** Reversal initiation and pumping inhibition triggered by optogenetic activation of AIB. Worms expressed ChR2 in AIB were challenged with varying intensities of blue light to tune the activity of AIB. Reversal initiation and pumping inhibition were plotted as a function of blue light intensities. Lower light intensities were required for triggering reversals. Error bars: s.e.m. *n* ≥ 10 worms

To further confirm the functional specificity of AIB neurons in transducing quinine sensory inputs to feeding inhibition, we kill AIB and other first layer interneurons (AIA, AIZ, and AIY) individually using both laser ablation and neuron-specific expression of apoptosis factor CED-3 (Fig. 2a). Both laser and chemical ablation of AIB, but not other interneurons, abolished quinine-induced feeding inhibition (Fig. 2b, c). Chemical ablation of AIB also blocked low-concentration quinine-induced reversal initiation (Supplementary Figure 2), consistent with its reported role in regulating reversal locomotion^15,21^. Interestingly, high-concentration quinine-induced reversals were only partially blocked by AIB ablation, suggesting a redundant circuit independent of AIB in initiating reversal locomotion.

If AIB indeed regulates both reversal initiation and feeding suppression as suggested by our neuron ablation experiments, acute stimulation of AIB should affect both behavioral outputs. To test this, we took an optogenetic approach by expressing the light-gated cation channel channel rhodopsin-2 (ChR2) specifically in AIB using the *npr-9* promoter^15,22^. As predicted, we found that the behavioral outputs stimulated by AIB activation were light strength-dependent. Namely, weak activation of AIB by low intensity (0.28 mW mm^−2^) laser illumination (488 nm) elicited reversal but not feeding suppression, whereas ~sixfold stronger laser illumination (1.77 mW mm^−2^) elicited both reversal and feeding suppression (Fig. 2d, e). Plotting behavioral outputs against stimulation light intensities clearly suggested distinct sensitivities of two different behaviors to AIB photoactivation (Fig. 2f), which further supports that higher intensity of sensory inputs on AIB elicits additional behavioral outputs (Fig.1). Taken together, the results of [Ca^2+^]_i_ imaging under native condition, neuron ablation and optogenetic experiments all strongly suggest that AIB acts downstream of ASH to mediate two quinine-induced behavioral outputs in a stimulus intensity-dependent manner: reversal initiation and feeding suppression. As locomotion and feeding are two types of behavioral outputs, we next sought to determine how different intensities of a single sensory input are decoded in AIB to produce this bifurcating pattern of commands to two behaviors.

### Glutamate induces reversal initiation and feeding suppression

**Fig. 3:**
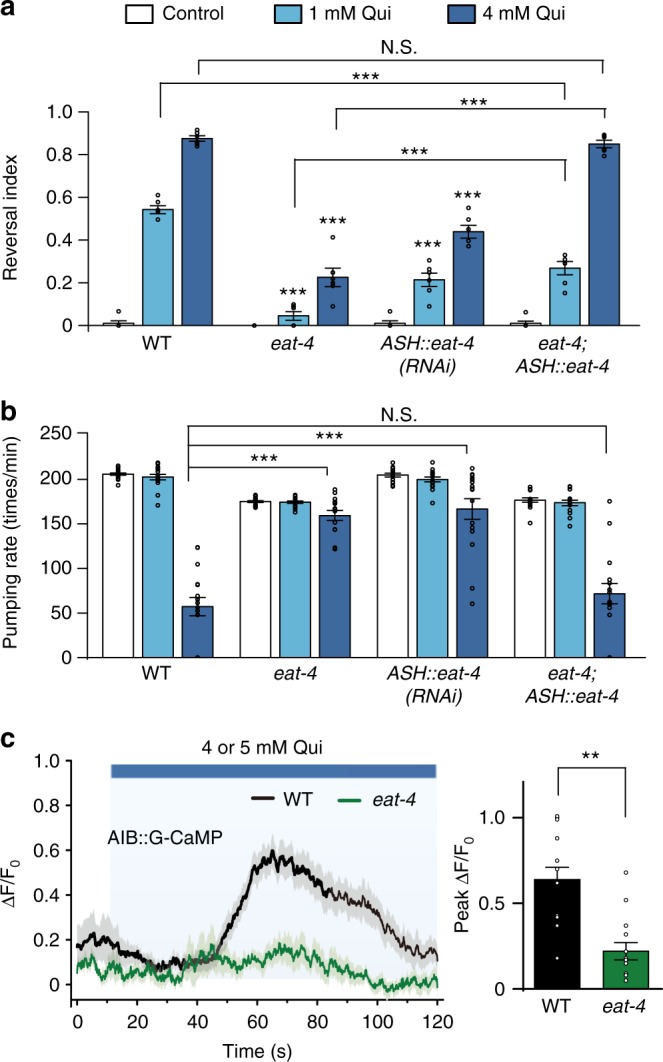
Glutamate signaling from ASH to AIB mediates both quinine-induced reversal initiation and feeding suppression. **a**, **b** Both reversal initiation and feeding suppression induced by quinine were abolished in *eat-4* mutant worms, and were dramatically declined in worms expressing *eat-4* RNAi as a transgene in ASH. Specific expression of wild-type *eat-4* gene in ASH partially rescued low-concentration quinine-induced reversal initiation, and fully restored high-concentration quinine-induced reversal initiation (**a**) and feeding suppression (**b**). Error bars: s.e.m. Reversal index, *n* = 6 groups, 10 worms/group at least; Pumping rate, *n* = 16 worms. ***p < 0.001 (*t* test or Wilcoxon test). **c** Calcium imaging shown that *eat-4* is required for quinine-induced calcium elevation in AIB. Left: average trace. The shades around traces indicate error bars (s.e.m.). *n* ≥ 14. **p < 0.01 (*t* test)

It has been reported that glutamate released from ASH is involved in nose touch-evoked reversal response^14,15^. Thus, we examined whether quinine-induced reversal locomotion and feeding suppression both require glutamate signaling from ASH to AIB. *eat-4* encodes a vesicular glutamate transporter, ablation of which causes deficiency in glutamatergic transmission^23,24^. We observed that both reversal initiation and feeding suppression induced by quinine were abolished in the *eat-4* mutants and were drastically reduced by ASH-specific RNAi of *eat-4* (Fig. 3). Neuron-specific expression of EAT-4 in ASH neurons partially rescued reversal initiation in response to low quinine concentrations and restored reversal initiation (Fig. 3a) and feeding suppression (Fig. 3b) in response to high concentrations of quinine. Furthermore, high-concentration quinine-induced [Ca^2+^]_i_ responses in AIB were substantially reduced in the *eat-4* mutants (Fig. 3c, Supplementary Figure 3). Taken together, these results suggest that both low and high-concentration quinine-induced behavioral outputs are dependent on glutamate signaling from ASH.

### GLR-1 and GLR-5 decode the intensity of quinine inputs

**Fig. 4:**
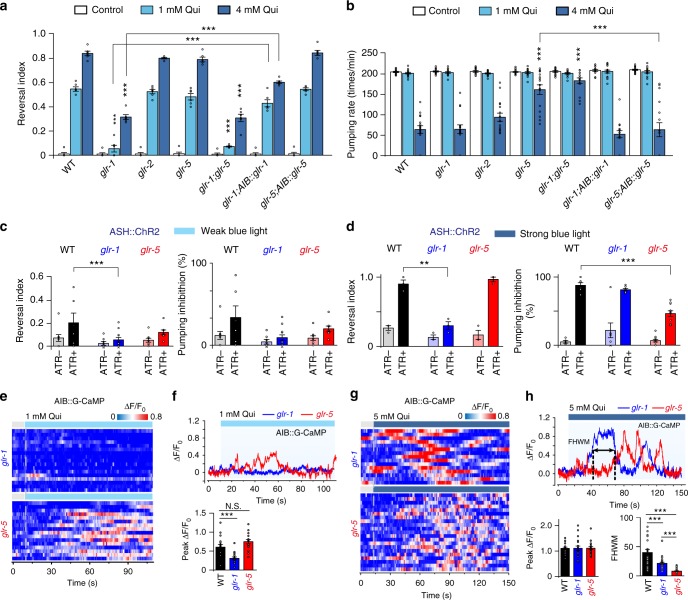
GLR-1 and GLR-5 in AIB are required for quinine-induced reversal initiation and pumping inhibition, respectively. **a** *glr-1* is required for quinine-induced reversal initiation, which can be rescued by transgenic expression of wild-type *glr-1* gene in AIB. Error bars: s.e.m. *n* = 6 groups, 10 worms/group. ***p < 0.001 (*t* test). **b** *glr-5* is required for quinine-induced pumping inhibition, which can be rescued by transgenic expression of wild-type *glr-5* gene in AIB. Error bars: s.e.m. *n* = 16 worms. ***p < 0.001 (*t* test or Wilcoxon test). **c** Optogenetic stimulation of ASH by blue light (470 nm; 0.03 mW mm^−2^; 30 s pulse) triggered reversals in wild-type worms, but not in *glr-1* mutant worms. The transgenic animals cultured on ATR-free plates were taken as control. **d** Optogenetic stimulation of ASH by blue light inhibited pumping rate in wild-type worms, but not in *glr-5* mutant worms. The transgenic animals cultured on ATR-free plates were taken as control. **c**, **d** Error bars: s.e.m.. Reversal index, *n* ≥ 3 groups, 10 worms/group; Pumping rate, *n* ≥ 5 worms. ***p < 0.001 (*t*- test). **e**, **g** AIB calcium dynamics induced by low (**e**) and high concentration of quinine (**g**), respectively, in freely behaving worms acquired by the iCaN system. Traces are ordered according to the time of quinine application (dash lines). **e**, **f** AIB calcium transients induced by 1 mM quinine were largely abolished in *glr-1* mutant worms. **e** Heat maps. **f** Representative traces (upper) and peak calcium changes (lower). Error bars: s.e.m.. *n* ≥ 16. ***p < 0.001 (*t*- test). N.S. represents no significant difference. **g**, **h** AIB calcium transients induced by 5 mM quinine in *glr-1* and *glr-5* mutant worms. **g** Heat maps. **h** Representative traces (upper), peak calcium changes and FHWMs for AIB calcium dynamics (lower). Notice that the peak amplitudes of AIB [Ca^2+^]_i_ elevations in neither *glr-1* nor *glr-5* mutant worms shown significant difference from wild-type worms, but the full width at half maximum (FWHM) were much longer in *glr-1* mutant worms than that in *glr-5*. Error bars: s.e.m. *n* ≥ 17. **p < 0.01. ***p < 0.001 (Wilcoxon test)

Next, we set to address how glutamate released from ASH could activate AIB. Three excitatory glutamate receptors, GLR-1, GLR-2, and GLR-5, and an inhibitory glutamate receptor AVR-14 have been shown to be expressed in AIB^23,25^. Our behavioral experiments showed that both low and high concentrations of quinine could initiate reversal in wild-type worms, *glr-2*, *glr-5*, and *avr-14* mutants, but not in *glr-1* mutant worms (Fig. 4a, Supplementary Figure 4). Moreover, AIB-specific rescue of GLR-1 significantly restored the reversal response to quinine in the *glr-1* mutant worms, confirming the action site of GLR-1 in AIB. On the other hand, *glr-5* mutants, but not other glutamate receptor mutants, showed defects in feeding suppression induced by high-quinine concentrations (Fig. 4b, Supplementary Figure 4). This defect could be largely rescued by restoring expression of GLR-5 in the AIB neuron of *glr-5* mutant background. Furthermore, *glr-1;glr-5* double mutant and *glr-1;glr-5;avr-14* triple mutant exhibited similar defects as *glr-1* and *glr-5* single mutants in reversal initiation and feeding suppression, respectively (Fig. 4a, b, Supplementary Figure 4). These results suggest that in response to ASH sensory inputs, GLR-1 and GLR-5, but not AVR-14, act independently in AIB neurons to mediate reversal initiation and feeding suppression, respectively.

To collect more evidence, we again employed optogenetic approach to stimulate ASH and record behavioral outputs under different strengths of stimulation and in different mutant background. We noticed that about 10 fold less power (0.031 mW mm^−2^) was required to excite ASH and initiate reversal (Fig. 4c) than that for AIB (Fig. 2). Importantly, reversal initiation was largely abolished in *glr-1* but not in *glr-5* mutants under both weak (Fig. 4c) and strong (Fig. 4d) ASH stimulation. In contrast, pumping suppression only occurred with strong ASH stimulation and was persisted in *glr-1* mutant but was significantly decreased in *glr-5* mutant (Fig. 4d).

To further illustrate the role of GLR-1 and GLR-5 in controlling AIB activity, we recorded quinine-induced [Ca^2+^]_i_ responses in AIB in the absence of either gene. As shown in Fig. 4e, f, low-concentration (1 mM) quinine-induced [Ca^2+^]_i_ elevations in AIB were largely abolished in the absence of GLR-1 but not GLR-5. At high quinine concentration (5 mM), whereas the peak amplitudes of [Ca^2+^]_i_ were comparable, the [Ca^2+^]_i_ elevations were sustained in *glr-1* but became transient in *glr-5* mutants (Fig. 4g, h). Analysis of the full width at half maximum (FWHM) of [Ca^2+^]_i_ elevations demonstrated longer FWHMs in *glr-1* than those in *glr-5* mutants (Fig. 4h). The [Ca^2+^]_i_ elevations were the longest in wild-type AIB neurons, probably due to an additive contribution of GLR-1 and GLR-5 to [Ca^2+^]_i_ signals. Furthermore, high-concentration (5 mM) quinine-induced [Ca^2+^]_i_ elevations in AIB were largely reduced in *glr-1*;*glr-5;avr-14* triple mutant, but not in *glr-1;avr-14* double mutant (Supplementary Figure 4c), suggesting that AVR-14 is not required for quinine-induced Ca^2+^ signaling in AIB. Together, these results suggest that GLR-1 and GLR-5 mediate different intensities of ASH sensory inputs on AIB and two patterns of [Ca^2+^]_i_ signals, transient and sustained, are differentially regulated by GLR-1 and GLR-5, respectively.

### Glutamate-evoked GLR-1 and GLR-5 currents in AIB neurons

**Fig. 5:**
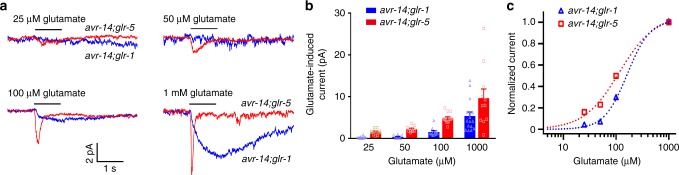
GLR-1 and GLR-5 currents gated by glutamate have different activation thresholds and kinetics. **a** Average traces of glutamate-evoked currents recorded in the AIB neurons of *avr-14;glr-1* and *avr-14;glr-5* double mutant background using perforated patch-clamp recording. A series of concentrations of glutamate (25 μM–1 mM) were perfused to the soma of AIB. AIB neurons were voltage clamped at −70 mV. *n* ≥ 5. **b** Quantification of the glutamate-induced currents in the AIB neurons of *avr-14;glr-1* and *avr-14;glr-5* double mutants, respectively. Error bars: s.e.m. *n* ≥ 5. **c** The normalized glutamate-induced currents in the AIB neurons of *avr-14;glr-1* and *avr-14;glr-5* double mutants were plotted as a function of the concentrations of glutamate. The tested glutamate concentrations were (in μM): 25, 50, 100, and 1000. Data were fit with the Hill equation: *I*/*I*_max_ = 1/[1 + (EC_50_/[glutamate])^*n*^], where *n* represents Hill slope (Hill coefficient)

Our above behavioral and calcium imaging experiments suggest that GLR-1 and GLR-5 act in AIB to trigger differential behaviors in response to different concentrations of quinine stimulation. Notably, both *glr-1* and *glr-5* encode putative orthologs of vertebrate non-NMDA glutamate receptors^26,27^. To understand how these two glutamate receptors decode the intensity of quinine inputs, we directly recorded glutamate-evoked electrical responses in the AIB neurons of dissected animals^19,26,28–30^. Since GLR-1, GLR-5, and AVR-14 glutamate receptors all act in AIB, we set to isolate GLR-1-mediated and GLR-5-mediated current in *avr-14;glr-5* and *avr-14;glr-1* double mutant background, respectively. To largely retain the intracellular contents, we recorded glutamate-induced currents in the AIB neurons using perforated patch-clamp recording. Interestingly, while low concentrations of glutamate induced a rapid activating and fast inactivating inward current in *avr-14;glr-5* double mutant (mediated by GLR-1), high concentrations of glutamate evoked a slow activating and more sustained inward current in *avr-14;glr-1* double mutant (mediated by GLR-5) (Fig. 5a, b). To further compare the glutamate sensitivity of GLR-1 and GLR-5, we plotted the normalized peak current amplitudes versus glutamate concentrations. As shown in Fig. 5c, glutamate-induced currents in *avr-14;glr-5* double mutant background were left-shifted compared to the ones obtained in *avr-14;glr-1* double mutant, implying that GLR-1 and GLR-5 have different sensitivities to glutamate. Notably, the reversal potentials of glutamate-induced currents in the AIB neurons of *avr-14;glr-5* and *avr-14;glr-1* double mutants were close to 0 mV, suggesting that both GLR-1 and GLR-5 are non-selective cation channels (Supplementary Figure 5). Furthermore, kainate (500 μM), but not AMPA, evoked similar currents in AIB to the ones induced by glutamate (Supplementary Figure 6), consistent with a previous observation in the AVA neuron^30^. Using a fosmid containing the full length segment of *glr-5* genomic DNA linked with GFP, we verified the expression of GLR-5 in AIB (Supplementary Figure 7). Taken together, our above results suggest a threshold-based mechanism in transmitting different intensities of sensory stimuli from ASH to AIB. In this model, as the quinine concentration increases it causes a gradual activation of ASH and thus a gradual increase in glutamate release. Post-synaptically, different levels of glutamate release are sensed by two glutamate receptors. Lower glutamate levels (i.e., that induced by 1 mM quinine) are sensed by the low-activation threshold GLR-1 receptor, while higher glutamate levels (i.e., that induced by 4–5 mM quinine) are sensed by the high-activation threshold GLR-5 receptor. The activation of these two glutamate receptors then triggers reversals and feeding inhibition, respectively.

### GLR-5 signals to Ca^2+^ stores

**Fig. 6:**
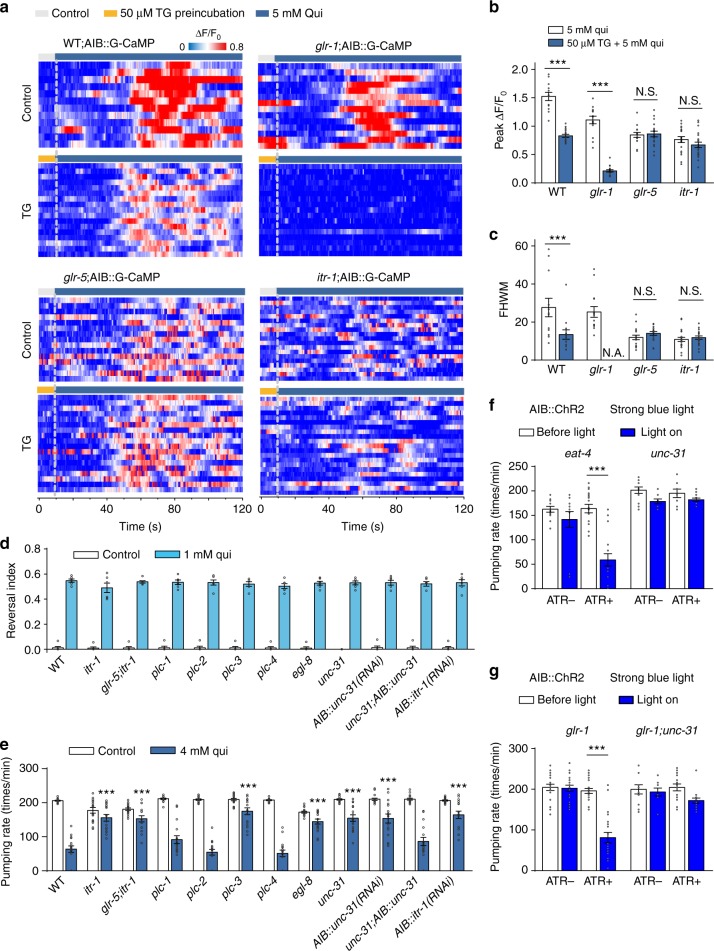
GLR-5 induces IP_3_-dependent Ca^2+^ release from intracellular Ca^2+^ store and triggers neuropeptide release. **a**, **b** AIB calcium dynamics induced by 5 mM quinine pre-treated with or without Thapsigargin (TG, treated for 3 min.) in freely behaving worms acquired by the iCaN system. Wild-type (WT), *glr-1*, *glr-5*, and *itr-1* mutant worms were tested. Traces are synchronized according to the time of quinine application (dash lines). **a** Heat maps; **b** peak calcium changes, and **c** FHWMs for AIB calcium dynamics. Error bars: s.e.m.. *n* ≥ 14. ***p < 0.001 (*t* test). N.S. represents no significant difference. **d**, **e** Quinine-induced feeding suppression (**d**), but not reversals (**e**), was dramatically defective in *egl-8*, *plc-3*, *itr-1*, and *unc-31* mutant worms. Error bars: s.e.m.. Reversal index, *n* = 6 groups, 10 worms/group at least; Pumping rate, *n* = 16 worms. ***p < 0.001 (*t* test). **e** Optogenetic stimulation of AIB inhibited pumping rate in *eat-4* mutant worms, but not in *unc-31* mutant worms. Worms-expressing ChR2 specifically in AIB were tested. The transgenic animals cultured on ATR-free plates were taken as control. Error bars: s.e.m. *n* ≥ 12. ***p < 0.001 (Wilcoxon test). **g** Optogenetic stimulation of AIB inhibited pumping rate in *glr-1* mutant worms, but not in *glr-1*;*unc-31* mutant worms. Worms expressing ChR2 specifically in AIB were tested. The transgenic animals cultured on ATR-free plates were taken as control. Error bars: s.e.m. *n* ≥ 12. ***p < 0.001 (Wilcoxon test)

Since the sensory inputs of quinine bifurcate in AIB through GLR-1 and GLR-5, we were curious as to how these two receptors drive two behavioral outputs from the same neuron. The presence of different [Ca^2+^]_i_ patterns in AIB (Fig. 4e–h) suggests that GLR-1 and GLR-5 activation may trigger Ca^2+^ signals from different sources. Namely, the GLR-1-induced [Ca^2+^]_i_ elevation is likely mediated, at least in part, by influx through the ionotropic GLR-1 channels^16,23^ themselves. In contrast, large, sustained [Ca^2+^]_i_ elevations are reminiscent of Ca^2+^ release from Ca^2+^ stores. We thus examined the potential involvement of intracellular Ca^2+^ stores in AIB glutamate signaling. We first employed a sarco-endoplasmic reticulum Ca^2+^-ATPase inhibitor (Thapsigargin, TG) which depletes the intracellular Ca^2+^ stores^31^. Upon TG (50 μM) treatment, the locomotion speed and pharyngeal pumping rate of worms were transiently inhibited and then recovered within 2 min. When we pre-treated *glr-1* mutant worms with TG, the high-concentration quinine-induced sustained [Ca^2+^]_i_ elevation in AIB was dramatically reduced, suggesting that the TG-sensitive Ca^2+^ stores are the major Ca^2+^ source for GLR-5-mediated Ca^2+^ signaling. By contrast, quinine-induced Ca^2+^ transients were unaffected by TG in *glr-5* mutant (Fig. 6a), excluding the involvement of TG-sensitive Ca^2+^ stores in the GLR-1-mediated Ca^2+^ signaling. Collectively, these results suggest that the sustained [Ca^2+^]_i_ elevations induced by high concentrations of quinine are largely due to Ca^2+^ release from Ca^2+^ stores downstream of GLR-5 activation.

Release of Ca^2+^ from intracellular stores may be controlled by IP_3_-mediated signaling acting through the IP_3_ receptor. In the absence of *itr-1* gene which encodes the *C. elegans* IP_3_ receptor, sustained [Ca^2+^]_i_ elevations in worms exposed to high-concentration quinine were diminished and became TG-insensitive, short [Ca^2+^]_i_ transients (Fig. 6a-c), suggesting that ITR-1 is required for high-concentration quinine-induced sustained [Ca^2+^]_i_ response. Consistent with this idea, quinine-induced feeding suppression, but not reversal, was also abolished in *itr-1* mutant (Fig. 6d, e). IP_3_ is produced by members of the phospholipase C (PLC) family. We found that quinine-induced feeding suppression was defective in mutants of two PLC genes, *egl*^*−*^*8* (PLCβ) *and plc-3* (PLCγ)^32^. Our above data implied similarities between the reversal duration and feeding suppression. Indeed, we verified that the reversal duration triggered by high concentrations of quinine was mainly impacted by *glr-5* and *itr-1* mutations, but not by *glr-1* mutation, suggesting that both the reversal duration and the pumping rate are controlled mainly by GLR-5 and intracellular Ca^2+^ stores (Supplementary Figure 8).

How does the interneuron AIB signal to its downstream neurons? *unc-31* encodes the ortholog of the mammalian CAPS protein, which has been shown to be specifically required for peptide release from dense core vesicles (DCVs)^33,34^. Interestingly, both the loss-of-function mutant of *unc-31* and AIB-specific *unc-31* RNAi abolished quinine-induced feeding suppression, which can be rescued by the expression of UNC-31 in AIB (Fig. 6d, e). To further confirm the release of DCVs from AIB, we optogenetically stimulated AIB by ChR2. As a control experiment, strong optogenetic activation of AIB elicited feeding suppression in wild-type, *eat-4* and *glr-1* mutants, indicating that EAT-4 and GLR-1 are not required for the AIB-mediated feeding suppression. In contrast, strong activation of AIB failed to suppress the pumping rate in either *unc-31* or *glr-1;unc-31* double mutants (Fig. 6f, g), supporting that the UNC-31-mediated DCVs release is required for the AIB-mediated feeding suppression. Taken together, these data strongly suggest the participation of Ca^2+^ store-regulated neuropeptide release from DCVs in GLR-5-mediated feeding suppression. Notably, our results that GLR-5 signaling is linked to Ca^2+^ stores are consistent with non-canonic coupling between vertebrate GIRK-1/GLUR5 and IP_3_-sensitive Ca^2+^ stores^35^.

### AIB suppresses feeding by activating RIM

**Fig. 7:**
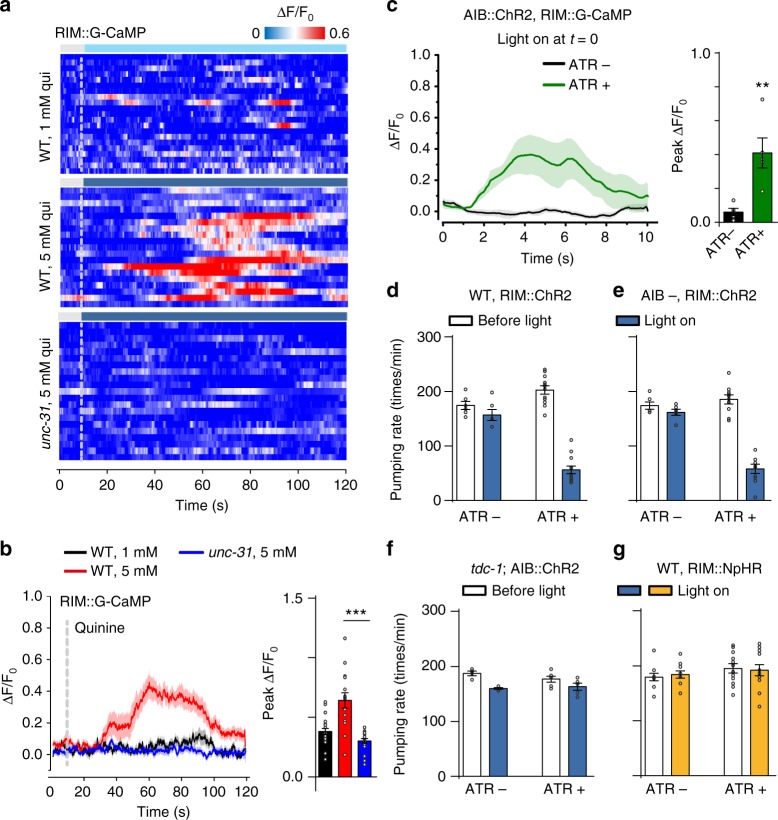
AIB suppresses feeding by activation of RIM via neuropeptides. **a**, **b** RIM calcium dynamics induced by quinine in freely behaving worms acquired by the iCaN system. Traces are synchronized according to the time of quinine application (dash lines). High-concentration quinine promoted the activity of RIM. Absence of *unc-31* abolished quinine-induced RIM activities. **a** Heat maps; **b** Average traces (left) and peak changes (right) for RIM calcium dynamics. The shades around traces indicate error bars (s.e.m.). *n* ≥ 15. ***p < 0.001 (*t* test). **c** Optogenetic stimulation of AIB promoted the activity of RIM. Worms expressing two transgenes (ChR2 in AIB, and GCaMP in RIM) were tested. The transgenic animals cultured on ATR-free plates were taken as control. Average traces (left) and peak changes (right) for RIM calcium dynamics were shown. The shades around traces indicate error bars (s.e.m.). *n* ≥ 5. **p < 0.01 (*t* test). **d**, **e** Optogenetic stimulation of RIM inhibited pumping rate in both wild-type and AIB-ablated worms. Worms expressing ChR2 specifically in RIM were tested. The transgenic animals cultured on ATR-free plates were taken as control. Error bars: s.e.m. *n* ≥ 6. ***p < 0.001 (*t* test). **f** *tdc-1* is required for RIM mediated pumping inhibition. *tdc-1* mutant worms expressing ChR2 specifically in RIM were tested. The transgenic animals cultured on ATR-free plates were taken as control. Error bars: s.e.m. *n* ≥ 4. N.S. represents no significant difference. **g** Optogenetic inhibition of RIM produced no effect on pumping. Worms expressing NpHR specifically in RIM were tested. The transgenic animals cultured on ATR-free plates were taken as control. Error bars: s.e.m. *n* ≥ 10. N.S. represents no significant difference

The *C. elegans* wiring diagram predicts bidirectional chemical synapses as well as gap junctions between AIB and RIM^4,8^. Previous studies have suggested an important role for RIM in reversals^13,15,20,22^. Here, we further investigated the connection between AIB and RIM during feeding suppression by monitoring Ca^2+^ levels in RIM. At low concentrations of quinine, we observed little activity in RIM. However, when challenged with high concentrations of quinine, we observed robust [Ca^2+^]_i_ elevations in RIM (Fig. 7a). Since ASH directly synapses to AVA and AVA is also electrically coupled with RIM^15^, it is possible that the signal from high concentrations of quinine could bypass AIB to activate RIM. To further confirm whether RIM acts downstream of AIB in feeding suppression, we carried out a number of experiments and obtained the following results: (1) Quinine-induced RIM activities were largely abolished in *unc-31* mutant (Fig. 7a, b), suggesting an essential role of neuropeptide release in the activation of RIM. (2) Strong activation of AIB by ChR2 elicited robust [Ca^2+^]_i_ elevations in RIM (Fig. 7c). (3) Direct activation of RIM by ChR2 inhibited pumping rate (Fig. 7d) even in the absence of AIB neurons (Fig. 7e). (4) In contrast to wild-type (Fig. 2e) and *eat-4* mutant (Fig. 6e), optogenetic activation of AIB in *tdc-1* mutant failed to inhibit pumping rate (Fig. 7f). Since *tdc-1* encodes a tyrosine decarboxylase required to convert tyrosine into tyramine in RIM^8,36^, AIB may act through tyramine to mediate feeding suppression. (5) Inhibition of RIM by a light-gated chloride pump halorhodopsin (NpHR) had no effect on pumping (Fig. 7g). Taken together, these results support that the activation of RIM by AIB is not only sufficient, but also essential in mediating feeding suppression.

## Discussion

Two opposing models have been proposed to explain how neural circuits decode stimulus intensity. According to the pattern theory, a stimulus of sufficient intensity elicits a distinct pattern across functionally indistinct sensory neurons; the resulting pattern is then decoded within the central nervous system to generate a specific behavior output, i.e., avoidance of noxious stimuli^37^. In contrast, the specificity theory posits that specific subtypes of sensory neuron are tuned to detect stimulus intensity via distinct receptors^38–40^. As such, information about the stimulus intensity is encoded, at least in part, by the primary sensory neuron itself, even before signals reach the interneurons. There are a number of examples which illustrate the specificity theory in action. For example, the sodium-selective ENaC channel acts as taste receptor for low-concentration salt and is required for behavioral attraction^41,42^, whereas high-concentration salt induces aversive behaviors which may involve two primary aversive taste pathways by activating the sour and bitter taste-sensing cells^1^. In this study, we have demonstrated a model where the stimulus intensity of quinine was encoded by differential signals flowing in the same neuronal connection. We interrogated the circuitry, synaptic and intracellular mechanisms underlying the decoding of quinine inputs by applying a multifaceted approach integrating Ca^2+^ imaging, optogenetics and behavioral analysis in freely moving animals together with genetic manipulation, laser and chemical ablation, and electrophysiology.

Our previous work revealed an ASH–RIM axis in the sensation of high-concentration quinine and feeding suppression^12^. However the synaptic connection between ASH and RIM remained to be explored. Here we conclude, based on four lines of evidence that AIB is the interneuron downstream of ASH in mediating feeding inhibition. First, [Ca^2+^]_i_ activities in AIB correlated with feeding inhibition (Fig.1). Second, ablation of AIB blocked quinine-induced feeding inhibition (Fig. 2). Third, optogenetic activation of AIB inhibited feeding (Fig. 2). Fourth, the identification of the presynaptic neurotransmitter glutamate from ASH and the post-synaptic receptor (GLR-5) on AIB to mediate feeding inhibition. Taken together, our results proposed a previously unidentified role for AIB in feeding regulation.

Interestingly, AIB is also known for its involvement in reversal initiation^15,16,21^. In this study, we confirmed that AIB is required for quinine-induced reversal initiation (Supplementary Figure 2). We showed that low concentration of quinine mediated AIB-dependent reversal, whereas high concentration of quinine-induced AIB-dependent reversal and feeding inhibition. How might changes in the intensity of sensory inputs to ASH result in additional behavior outputs through the same AIB interneuron? One interesting feature of *C. elegans* neurons is that they are non-spiking and activated in a graded manner^43^. Different strength of stimulation of ASH is therefore likely to result in graded glutamate release, which could be decoded in AIB via receptors with different activation threshold and different kinetics for glutamate. Indeed, using single-neuron electrophysiology, we proved that GLR-1 has a lower activation threshold and fast kinetics for glutamate, while GLR-5 has a higher activation threshold and more sustained kinetics (Fig. 5). Hence, a single-analog sensory input generates graded response at ASH, which is then bifurcated at the AIB interneuron via differential responses of GLR-1 and GLR-5 receptors.

GLR-1 belongs to the non-NMDA-type ionotropic glutamate receptor family which can be activated by either glutamate or kainate, and regulates a broad range of physiological functions^15,23,26,28^. It has been suggested that GLR-1 mediates rapid activating and inactivating currents and that Ca^2+^ permeation through GLR-1 causes an increase in [Ca^2+^]_i_ of post-synaptic targets^23,28^. In contrast, neither the biophysical and pharmacological properties nor the physiological function of GLR-5 has been reported before. Mammalian GluK1/GluR5 is a kainate-type ionotropic glutamate receptor subunit, which is an important mediator of the pre- and post-synaptic actions of glutamate and has been linked to a number of brain disorders such as epilepsy, schizophrenia, and autism^29,44^. In addition to their channel-mediated effects, kainate receptors can signal through an unconventional metabotropic mechanism involving G-proteins and second messengers^44^. Interestingly, it has been demonstrated that kainate can induce a G protein-dependent rise in [Ca^2+^]_i_ via the Ca^2+^ release from IP_3_-sensitive Ca^2+^ stores^35,44,45^ and can act through PLC^32,44^. In this study, we demonstrated for the first time a GLR-5-dependent excitatory response in AIB (Fig. 5). Similar to GLR-1, GLR-5 can be activated by both glutamate and kainate (Fig. 5, Supplementary Figure 6). Activation of GLR-5 induced a large and long-lasting [Ca^2+^]_i_ increase that requires Ca^2+^ release from intracellular stores and components of the IP_3_-mediated Ca^2+^ signaling pathway, i.e., PLC and the IP_3_-receptor (ITR-1 in *C. elegans*).

AIB forms unusually dense synaptic connections to RIM with over 30 synapses between the two neurons^4^. The valence and temporal properties of links between AIB and RIM are ambiguous. Previously it has been suggested that AIB-mediated inhibition of RIM activity during reversals^13,15^. Actually the correlation of activities between AIB, RIM, and AVA is complicated due to the complex forward and backward chemical synapses and gap junctions between these neurons, which form a built-in variability generator triggering random reversals^20^. In contrast to the inhibitory transmission between AIB and RIM, we identified an excitatory connection of AIB–RIM in response to high-concentration quinine. How could a single synapse behave as both inhibitory and excitatory? We think this discrepancy can be reconciled by the differential response to different strength of sensory inputs. At low strength of ASH input, i.e., gentle nose touch or low concentration of quinine, AIB might inhibit RIM via the glutamate-gated Cl^−^channel AVR-14^15^. Inhibition of RIM then allows activation AVA and triggers reversal via the so-called disinhibitory circuit^15^. At high concentration of quinine, glutamate release from both ASH and AIB can activate AVA in parallel and initiate reversal as previously suggested^15^. On the other hand, high-concentration quinine also triggers the release of neuropeptide-containing DCVs, which are distant from Ca^2+^ channels and more sensitive to Ca^2+^ release from intracellular Ca^2+^ stores^46^. Neuropeptide release from AIB then excites RIM and induces tyramine-dependent feeding inhibition. In Supplementary Figure 9, we summarized the disinhibitory circuit for reversal (dashed line) and the AIB–RIM excitation circuit for feeding regulation. In fact, our previous results also suggest an inhibition of RIM at normal condition, where RIM receives inhibitory inputs from NSM and is largely locked in an inhibitory state in a “flip-flop” circuit^12^. Hence, the activation of RIM requires strong input, i.e., neuropeptide, to overcome the combined inhibition of 5-HT from NSM^12^ and glutamate from AIB^15^. Activation of RIM at strong sensory input can activate AVA through gap junctions^20,22^ and is thus correlated with long-lasting reversals (Fig. 1d, e).

One of the most fundamental question in neuroscience is how single neurons encode complex behavioral outputs. A recent study has identified an interesting strategy by which one interneuron, AIY, regulates two distinct behavioral outputs: locomotion speed and direction switch^47^. This strategy employs the same neurotransmitter ACh but utilize distinct post-synaptic ACh receptors on different neurons (RIB and AIZ) to command speed and direction switch, which is more akin to specificity theory. In contrast, the current study reveals a different strategy, more like a pattern theory, where distinct signal patterns are employed in the same neurons. At the ASH–AIB synapse, graded presynaptic glutamate signal is decoded and diverges by activating two post-synaptic glutamate receptors in the same AIB neuron. In AIB interneuron, two distinct Ca^2+^ patterns were employed to induce different neurotransmitter release, glutamate and neuropeptides. At the AIB–RIM synapse, the valence can be either inhibitory or excitatory depending on the neurotransmitter released from AIB (Supplementary Figure 9). This design might have integration advantages since behaviors are encoded in the same neuron by different intracellular signals.

Apparently, the neuron specificity^47^ and signal specificity (this study) strategies complement each other and contribute to the multifunctional properties of neural circuits. There is still much to learn about how the nervous system decodes and encodes different behavioral outputs. Nevertheless, our findings highlight the importance of determining the valence, strength, and temporal properties at the single-neuron level during connectome construction. Furthermore, our analysis of specific avoidance behaviors in *C. elegans* may help uncover the mechanisms of polymodal signaling such as nociception in more complex organisms since the involvement of neuropeptides in nociception is highly conserved from worms to mammals^48,49^.

## Methods

### Correlated Ca^2+^ imaging, optogenetics and behavioral tracking

We developed a fast tracking system iCaN (Imaging Calcium activities of Nematodes), as described in Supplementary Figure 1, to simultaneously monitor and manipulate neural activities while analyzing locomotion and feeding behaviors in a freely moving worm. Worms were cultured on Nematode growth medium (NGM) plates at 20 ℃ with *E. coli* OP50 using standard procedures. Well-fed young adult hermaphrodites were randomly selected and used for all experiments unless otherwise mentioned. A full list of strains used in this study is available in the Supplemental Methods.

Neuronal calcium activity was measured by detecting changes in fluorescence of GCaMP, a genetically encoded calcium indicator. Fluorescence of mKate2 was used as reference in most experiments. Neuronal activities were manipulated optically by illuminating targeted neurons expressing the optogenetic protein ChR2 or NpHR^15,22^.

In general a worm was transferred to a small piece of agar spread with a thin layer of OP50 bacteria. The agar pad was prepared with NGM without cholesterol. Briefly, five layers of sticky labels (thickness measured as 0.5 mm) were pasted on the edge of a glass slide. A droplet of liquid agar was added on the slide before sandwiching with another glass slide. As a result, the agar layer has a thickness of 0.5 mm (different thicknesses of agar pad could be prepared by changing the layers of sticky labels). Next, we cut this agar pad into a 10×10 mm^2^ right before a worm was transferred onto the pad. The worm was then covered carefully with a cover slip and viewed under the microscope with cover slip down. Five microlitre aliquots of quinine stock solution were dropped on the top of the agar mount and allowed to permeate through agar to reach the worm. To calculate the delivered stimulus intensity, the amount of quinine was divided by the volume of agar. It should be noted that the 20–30 s delay of [Ca^2+^]_i_ response to quinine stimuli is due to the diffusion of quinine through the thick agar pad (0.5 mm) because the lag between ASH and AIB response is quite short (Supplementary Figure 10). Indeed, if we reduced the thickness of agar to one page of sticky label (0.1 mm), we obtained much faster [Ca^2+^]_i_ responses in ASH and AIB (Supplementary Figure 10). To define [Ca^2+^]_i_ peaks, the 10 s baseline response before quinine stimulation was averaged as basal [Ca^2+^]_i_ and the maximal increase of [Ca^2+^]_i_ level after quinine stimulation was defined as the [Ca^2+^]_i_ peak response. If multiple [Ca^2+^]_i_ peaks are present in one cell, we selected the largest one as the peak response.

For optogenetic experiments, worms were cultured on NGM plates supplied with OP50 and 5 mM all-trans retinal (ATR) and the same transgenic animals cultured on ATR-free plates were used as control. All imaging experiments and optogenetic manipulations were performed on *lite-1(xu7)* or *lite-1(ce314)* genetic background to eliminate the intrinsic photophobic response^50^. Illumination was applied by blue light ~488 nm in 30 s pulses. For AIB activation, strong blue light was about 1.77 mW mm^−2^ and weak blue light was 0.28 mW mm^−2^. For ASH activation, the light power was 0.2 mW mm^−2^ and 0.03 mW mm^−2^, respectively.

Pumping rate and locomotion were monitored by the iCaN system and assayed using a custom-developed software as described in Supplemental Methods.

Cross-correlation analyses were performed with Origin Lab Pro.9.0.

### Laser ablation

Laser ablation was performed on L1 or L2 stage worms by standard procedures^51^. A pulsed laser was used to ablate target neurons. Ablation was confirmed the following day and worms were used for experiments as young adults.

### Electrophysiology

Electrophysiological recordings were carried out on an Olympus microscope (BX51WI) with an EPC-10 amplifier and Patchmaster software (HEKA). Day 2 adult worms were glued on the surface of Sylgard-coated coverslips using cyanoacrylate-based glue. A dorsolateral incision was made using sharp glass pipettes to expose the AIB neurons marked by GCaMP3.0 and mKate2^29,30,50^. The bath solution contained (in mM) 145 NaCl, 2.5 KCl, 5 CaCl_2_, 1 MgCl_2_, 20 glucose (325–335 mOsm, pH adjusted to 7.3). The intracellular solution (ICF) contained (in mM) 145 K-gluconate, 5 MgCl_2_, 5 EGTA, 0.25 CaCl_2_, 10 HEPES, 10 glucose, 5 Na_2_ATP, and 0.5 NaGTP (315–325 mOsm, pH adjusted to 7.2). High Cl^−^ ICF: same as ICF with the following changes: 145 mM KCl. For perforated recording, 120 µg mL^−1^ of nystatin was included in ICF. Membrane potential was clamped at −70 mV. Glutamate and kainate were diluted in bath solution and perfused toward the soma of the AIB neuron using a MINJ-D Digital Positive Displacement System (Tritech).

### Statistical analysis

Data are expressed as the mean ± s.e.m., and the statistical significance of differences was assessed using the two-sided *t* test when the data meet the assumptions of the *t* test. If the data did not meet the normal distribution, the Wilcoxon test was used. The sample sizes were determined by the reproducibility of the experiments and are similar to those generally employed in the field.

## Electronic supplementary material

Supplementary Information

## Data Availability

iCaN system used a java-based custom software. Pharyngeal pumping rate (feeding behavior) was quantified using a java-based custom software. The codes are available from the authors upon request. All relevant data are available from the authors upon request.
