# Supplementary material for: Decoding the intensity of sensory input by two glutamate receptors in one C. elegans interneuron
Source: Nat Commun. 2018 Oct 17;9:4311. doi: 10.1038/s41467-018-06819-5 (PMC6193023; doi:10.1038/s41467-018-06819-5)
Supplement: Supplementary file 1 — Supplementary Information [file 41467_2018_6819_MOESM1_ESM.pdf]

Supplementary Information

Decoding the intensity of sensory input by two glutamate receptors in one *C. elegans* interneuron

Zou et al.

Supplementary Methods

Nematode strains

The following worm strains were used:

WT: N_2 (the wild type).
KG1180: *lite-1*(*ce314*).
MT6308: *eat-4*(*ky5*).
KP4: *glr-1*(*n2461*).
VM4314: *glr-1*(*ky176*).
RB1808: *glr-2*(*ok2342*).
TM3506: *glr-5*(*tm3506*).
DA1371: *avr-14(ad1032)*.
JT73: *itr-1(sa73)*.
glr-1(*n2461*);*glr-5*(*tm3506*).
avr-14(ad1032);glr-1(*n2461*).
avr-14(ad1032);*glr-5*(*tm3506*).
glr-1(*n2461*);*glr-5*(*tm3506*);*avr-14(ad1032)*.
glr-5(tm3506);itr-1(sa73).
CB928: *unc-31*(*e928*).
TM753: *plc-1(tm753)*.
RB1496: *plc-2(ok1761)*.
TM1340: *plc-3(tm1340).*
RB1173: *plc-4(ok1215)*.
CB6614: *elg-8*(*e2917*).
TQ738: *lite-1* (*ce314*);xuEx11[*sra-6*::ChR2::YFP].
TQ1914: *lite-1*(*xu7*);xuIs28[*npr-9*::ChR2::YFP, *npr-9*::DsRed2, *unc-122*::GFP].
TXL180: *lite-1*(*ce314*);txuEx180[*npr-9*::GCaMP3, *npr-9*::mKate2, *lin-44*::GFP].
TXL181: *glr-1*(*n2461*);*lite-1*(*ce314*);txuEx181[*npr-9*::GCaMP3, *npr-9*::mKate2, *lin-44*::GFP].
TXL182: *glr-2*(*ok2342*);*lite-1*(*ce314*);txuEx182[*npr-9*::GCaMP3, *npr-9*::mkate2, *lin-44*::GFP].
TXL183: *glr-5*(*tm3506*);*lite-1*(*ce314*);txuEx183[*npr-9*::GCaMP3, *npr-9*::mKate2, *lin-44*::GFP].

TXL184: *glr-1*(*n2461*);*glr-5*(*tm3506*);*lite-1*(*ce314*);txuEx184[*npr-9*::GCaMP3, *npr-9*::mkate2, *lin-44*::GFP].

TXL185: *glr-5*(*tm3506*);txuEx185[*npr-9*::*glr-5*::SL2::mKate2, *vha-6*::mKate2].

TXL186: *eat-4*(*ky5*);*lite-1*(*ce314*);txuEx186[*npr-9*::GCaMP3, *npr-9*::mKate2, *lin-44*::GFP].

TXL187: *lite-1*(*xu7*);xuIs28[*npr-9*::ChR2::YFP, *npr-9*::DsRed2, *unc-122*::GFP]; txuEx187[*npr-9*::CED-3, *vha-6*::GFP].

TXL188: *lite-1*(*ce314*);txuEx188[*sra-6*::GCaMP2::SL2::mKate2, *npr-9*::GCaMP3, *npr-9*::mKate2, *lin-44*::GFP].

TXL189: *lite-1*(*ce314*);xuEx11[*sra-6*::ChR2::YFP];txuEx180[*npr-9*::GCaMP3, *npr-9*::mKate2, *lin-44*::GFP].

TXL190: txuEx190[*sra-6*::*eat-4RNAi*::SL2::mKate2, *vha-6*::mKate2].

TQ1077: txuEx[*gcy-13*::NpHR::YFP, *gcy-13*::DsRed2, *unc-122*::GFP].

TXL191: *itr-1(sa73)*;*lite-1*(*ce314*);txuEx191[*npr-9*::GCaMP3, *npr-9*::mKate2, *lin-44*::GFP].

TXL192: *eat-4(ky5)*;txuEx192[*npr-9*::*eat-4*::SL2::mKate2, *lin-44*::GFP].

TQ2881: *glr-1(n2461);*xuEX[*npr-9*::*glr-1cDNA*::SL2::YFP, *npr-9*::DsRed, *unc-122*::RFP].

TQ2905: *eat-4(ky5)*;xuEX[*sra-6*::*eat-4cDNA*::SL2::YFP, *npr-9*::Dsred, *unc-122*::RFP].

TQ1866: *lite-1(ce314)*;xuEx308[*cex-1*::GCaMP1.3, *cex-1*::mCherry2].

TXL193: *lite-1(ce314)*;xuEx308[*cex-1*::GCaMP1.3, *cex-1*::mCherry2]; xuIs28[*npr-9*::ChR2::YFP, *npr-9*::DsRed2, *unc-122*::GFP].

TXL194: *lite-1*(*ce314*);txuEx194[*cex-1*::ChR2::YFP, *lin-44*::GFP].

TXL195: *tdc-1(n3419)*;*lite-1(ce314)*;xuIs28[*npr-9*::ChR2::YFP, *npr-9*::DsRed2, *unc-122*::GFP].

TXL196: *eat-4(ky5)*;*lite-1(ce314)*;xuIs28[*npr-9*::ChR2::YFP, *npr-9*::DsRed2, *unc-122*::GFP].

TXL197: *lite-1*(*ce314*);xuIs28[*npr-9*::ChR2::YFP, *npr-9*::DsRed2, *unc-122*::GFP]; txuEx187[*npr-9*::CED-3, *vha-6*::GFP];txuEx197[*tdc-1*::ChR2::GFP, *lin-44*::GFP].

TXL198: txuEx198[*npr-9*::*itr-1RNAi*::SL2::mKate2, *lin-44*::GFP].

TQ1252: xuEx21[*npr-9*::YFP, *npr-9*::DsRed, *unc-122*::GFP].

TQ5260: *lite-1(ce314);*xuEx1698[*sto-3s*::mCherry + *ttx-3s*::sl2::YFP].

TQ3041: *lite-1(xu7);*xuEx1042[*ser-2(2)*::frt::GFP, *odr-2(2b)*::FLP, *unc-122*::GFP].

TQ3051: xuEx1046[*gcy-28d*::sl2::YFP].

TXL199: *glr-1*(*n2461*);*lite-1*(*ce314*);xuEx11[*sra-6*::ChR2::YFP].

TXL200: *glr-5*(*tm3506*);*lite-1*(*ce314*);xuEx11[*sra-6*::ChR2::YFP].

TXL201: *unc-31*(*e928*);*lite-1(ce314)*;xuIs28[*npr-9*::ChR2::YFP, *npr-9*::DsRed2, *unc-122*::GFP].

TXL202: *lite-1(ce314)*;txuEx202[*cex-1*::GCaMP3::SL2::mKate2, *lin-44*::GFP].
TXL203: *unc-31*(*e928*);*lite-1(ce314)*;txuEx202[*cex-1*::GCaMP3::SL2::mKate2, *lin-44*::GFP].
TXL204: *lite-1*(*ce314*);xuIs28[*npr-9*::ChR2::YFP, *npr-9*::DsRed2, *unc-122*::GFP]; txuEx187[*npr-9*::CED-3, *vha-6*::GFP];xuEx308[*cex-1*::GCaMP1.3, *cex-1*::mCherry2].
TXL205: *avr-14(ad1032)*;*glr-1*(*n2461*);*lite-1*(*ce314*);txuEx181[*npr-9*::GCaMP3, *npr-9*::mKate2, *lin-44*::GFP].
TXL206: *avr-14(ad1032)*;*glr-1*(*n2461*);*glr-5*(*tm3506*);*lite-1*(*ce314*);txuEx184[*npr-9*::GCaMP3, *npr-9*::mkate2, *lin-44*::GFP].
TXL207: txuEx184[*glr-5* fosmid, *npr-9*::mkate2, *lin-44*::GFP].
Kan3010: *glr-1*(*n2461*);*lite-1(ce314)*; xuIs28[*npr-9*::ChR2::YFP, *npr-9*::DsRed2, *unc-122*::GFP].
Kan3011: *glr-1*(*n2461*);*unc-31*(*e928*);xuIs28[*npr-9*::ChR2::YFP, *npr-9*::DsRed2, *unc-122*::GFP].
Kan3890: *avr-14(ad1032);glr-1*(*n2461*);kanEx281[*npr-9*::mkate2, *lin-44*::GFP].
Kan3891: *avr-14(ad1032)*;*glr-5*(*tm3506*);kanEx281[*npr-9*::mkate2, *lin-44*::GFP].

Molecular Biology

Promoters labeling specific neurons are described as below: AIB: *npr-9*, RIM: *gcy-13* or *cex-1*, ASH: *sra-6*. The promoters were PCR-amplified from N2 genomic DNA. These PCR fragments were recombined with the Hind III and BamH I, Sph I and BamH I, Xba I and BamH I sites in the modified donor vectors, respectively. In this way, AIB::GCaMP3.0, AIB::ChR2, RIM::GCaMP1.3, and ASH::GCaMP3.0 plasmids were generated. Neuron-specific RNAi was generated as previously described[1]. The *itr-1* RNAi segment was amplified with the primers 5'-CAAGGCTCTTCGGAACTTCATT and 5'-CAGAATGTGATCAACATATTCTGTT. The *eat-4* RNAi segment was amplified with the primers 5'-GAAGGAAACGAAAACCCGATGC and 5'-CCACAACTCCAGAGGATCCATG from N2 cDNA.

The expression pattern of *glr-5* was conducted by a fosmid from Source BioScience with Clone Transgene Ome Resource: CBGtg9050H043D, which contain the full length segment

of *glr-5* and about 15 kb upstream and 10 kb downstream sequence, attached with a GFP sequence before its stop codon. This fosmid was co-injected with a plasmid *Pnpr-9*::mkate2, which is specifically expressed in AIB neuron.

Behavioral Assays for Reversal

To calculate reversal index, a previously described protocol of the 'drop test' assay is employed[2]. In brief, a drop of quinine solution was delivered near the tail of a forward-moving animal. When the repellent quinine was concentrated enough, the animal ceases forward movement and reverses; if quinine was diluted sufficiently, extremely just water alone, the animal will continue moving forward. The response of a single animal to each drop delivered was recorded as either a positive or a negative response. Response was considered positive when the avoidance reflex was observed within 4 s after the animal encountered quinine. The results of the reversal assays were represented as a reversal index, which was defined as the number of positive responses divided by the total number of trials (drops delivered). At least 10 worms a group were tested to calculate a reversal index, and more than 6 groups were tested per experiment. Every experiment was repeated at least three times. The assay was conducted always on unseeded NGM plates. An interval of 10 min was used between successive drops challenged to the same animal. Each animal was tested with no more than 5 successive drops per day.

Supplementary Figures and Figure Legends

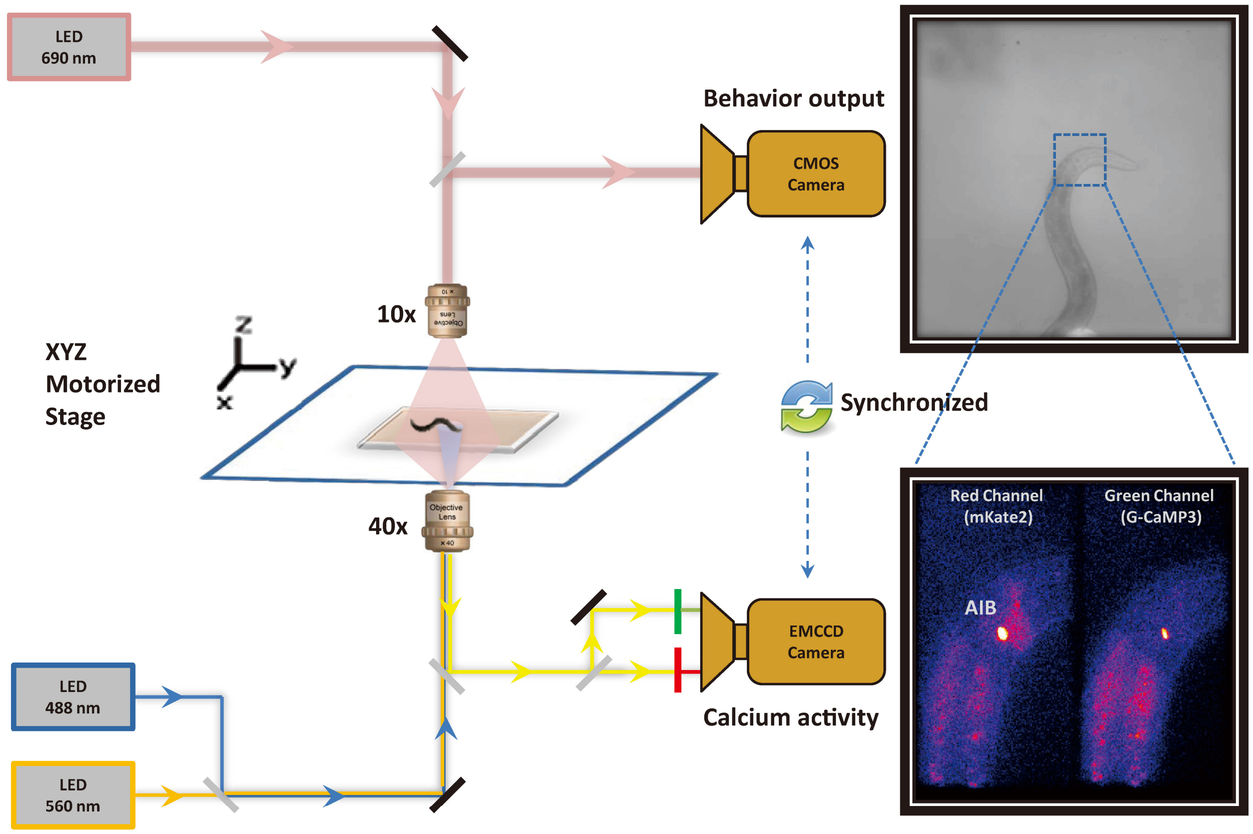

Supplementary Figure 1. Simultaneous neuronal calcium imaging, optogenetics and behavioral tracking in freely moving worms.

A schematic drawing of the iCaN system, which was developed to simultaneously analyze the locomotion and feeding behaviors while monitoring intracellular Ca^{2+} concentration ($[Ca^{2+}]_i$) at single neuron resolution on freely moving worms. A low-magnification objective on top of the sample was used to track the movement of free-moving worms and to record the feeding behavior. A high-magnification objective from the bottom to monitor the neuronal $[Ca^{2+}]_i$ activities at the single neuron resolution. The recorded locomotion is feedback to x-y stage to center the worm in the field-of-view of both objectives. Special z stacking design is undertaken to ensure fast 4D imaging of worm neurons at high resolution. Changes in fluorescence of the calcium indictor GCaMP were monitored with an

EMCCD camera. Fluorescence of mKate2 was acquired as reference in most experiments. To simultaneously monitoring GCaMP fluorescence and turning on ChR2 or NpHR, a LED light source emitting blue and yellow light was added to the iCaN system. Pharyngeal pumping rate (feeding behavior) was quantified using a custom-developed software. Cross-correlation analyses were performed with Origin Lab Pro.9.0.

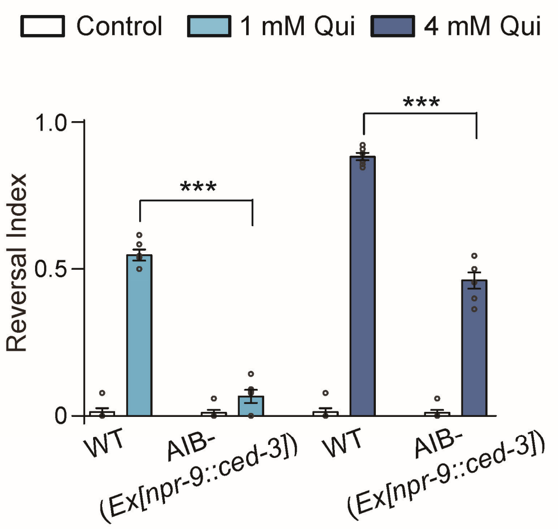

Supplementary Figure 2. AIB is required for reversal initiation.

Chemical deletion of AIB significantly suppressed reversals induced by both low and high concentrations of quinine, respectively. CED-3 was expressed specifically in AIB. Error bars: s.e.m. n = 6 groups, 10 worms/group at least. ***p < 0.001 (t test).

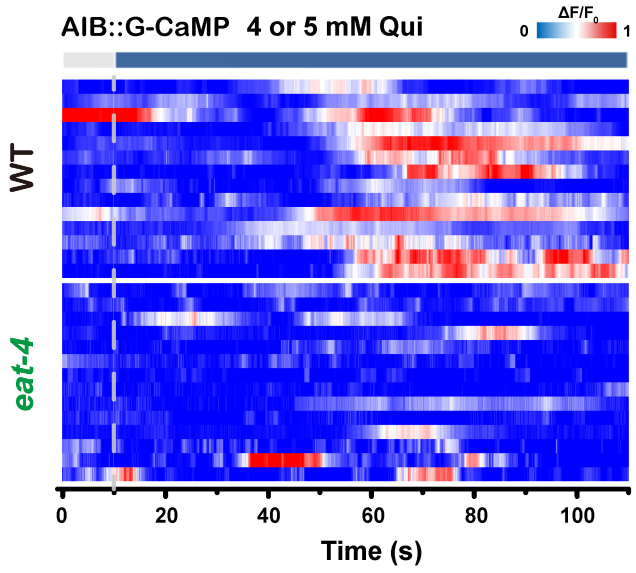

Supplementary Figure 3. Glutamate signaling is essential for high concentration quinine-induced $[Ca^{2+}]_i$ elevation in AIB.

Heat maps showing AIB calcium dynamics induced by 4 or 5 mM quinine in wild-type and *eat-4* mutant worms, respectively.

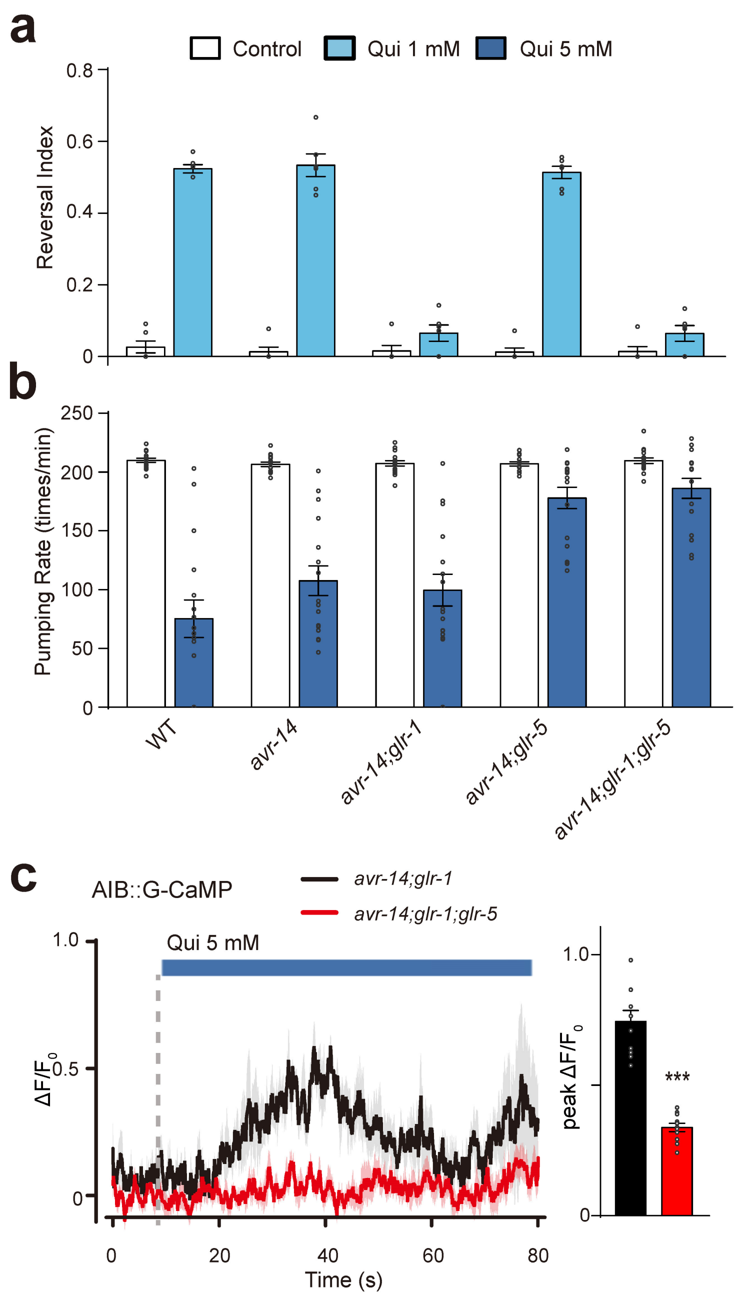

Supplementary Figure 4. AVR-14 is not required for quinine-induced reversal initiation and pumping inhibition.

(a) *avr-14* is not required for quinine-induced reversal initiation. Error bars: s.e.m.. n = 6 groups, 10 worms/group at least.

(b) *avr-14* is not required for quinine-induced pumping inhibition. Error bars: s.e.m.. n = 16 worms.

(c) AIB calcium transients induced by 5 mM quinine in *avr-14;glr-1 double mutants* and *avr-14;glr-1;glr-5* triple mutants, suggesting the requirement for both GLR-1 and GLR-5, but not

AVR-14. Left: Average trace. The shades around traces indicate error bars (s.e.m.). Right: Peak calcium changes. n ≥ 5. ***p < 0.001 (t test).

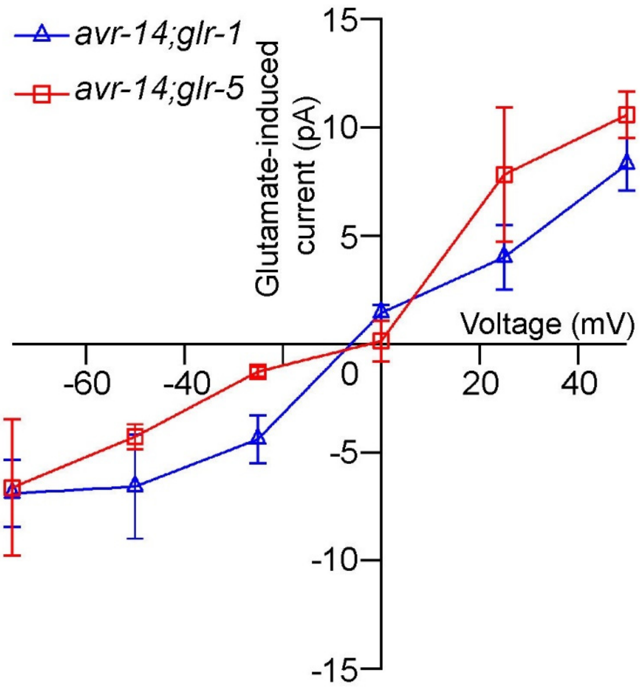

Supplementary Figure 5. I-V relationships of glutamate-induced currents in AIB.

Quantification of currents in AIB induced by 1 mM glutamate in *avr-14;glr-1* and *avr-14;glr-5* double mutant worms. AIB neurons were voltage clamped at -75, -50, -25, 0, 25 and 50 mV. Error bars: s.e.m. n ≥ 2.

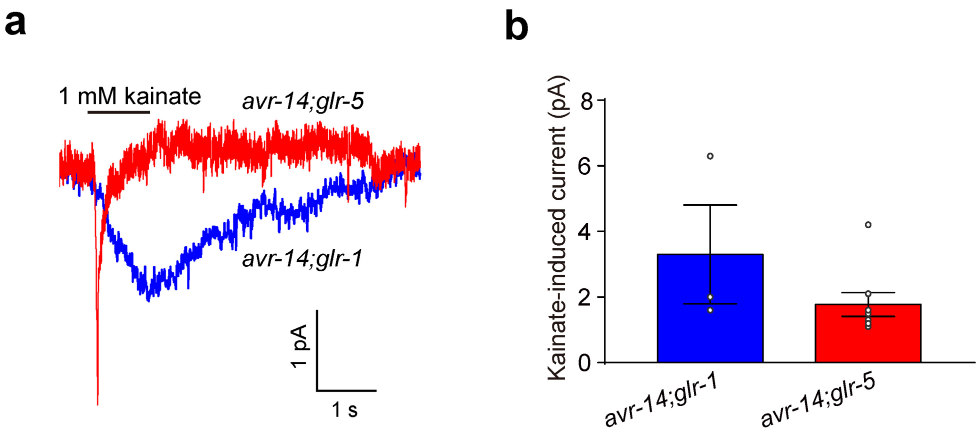

Supplementary Figure 6. GLR-1 and GLR-5 have distinct kinetics to kainate.

(a) Average traces of kainate-evoked currents recorded in AIB using perforated patch-clamp recording. *avr-14;glr-1* double mutant and *avr-14;glr-5* double mutant worms were tested. The AIB neurons were voltage clamped at -70 mV. n ≥ 3.

(b) Quantification of currents in AIB induced by 0.5 mM kainate in *avr-14;glr-1* double mutant and *avr-14;glr-5* double mutant worms. Error bars: s.e.m. n ≥ 3.

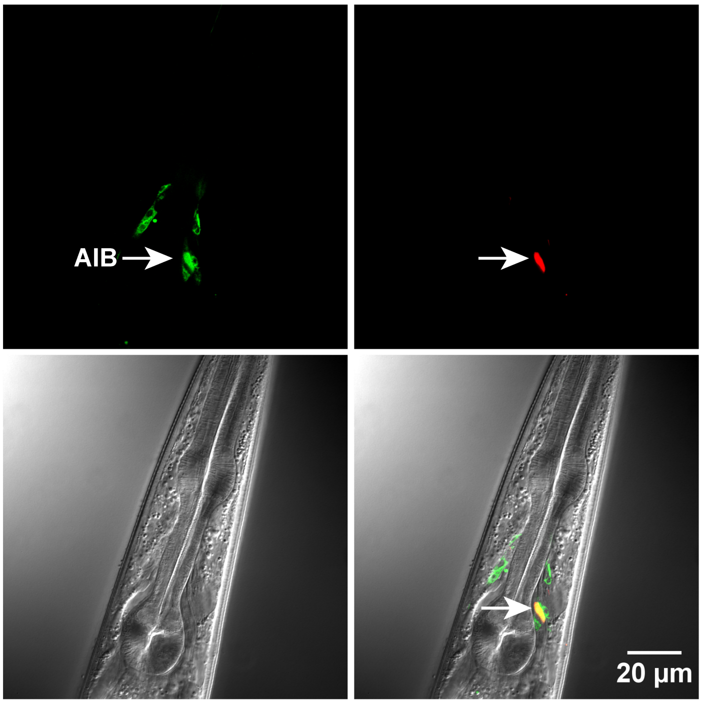

Supplementary Figure 7. The expression of GLR-5 in AIB.

Fosmid containing the full length segment of *glr-5* attached with GFP was co-injected with a plasmid P*npr-9*::mkate2 which is specifically expressed in AIB. Images captured by confocal microscopy confirmed the expression of *glr-5* in AIB. The arrows point to AIB.

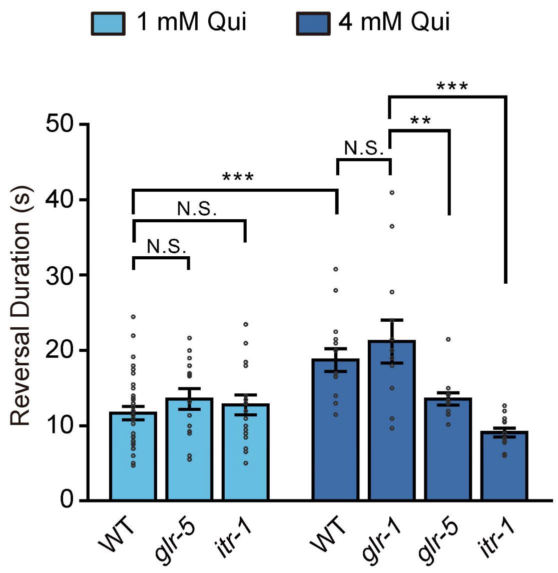

Supplementary Figure 8. The duration of reversal triggered by high concentrations of quinine is mainly controlled by GLR-5 and intracellular Ca^{2+} stores.

Error bars: s.e.m. n ≥ 12. N.S., not significant, **p < 0.01, ***p < 0.001 (t test).

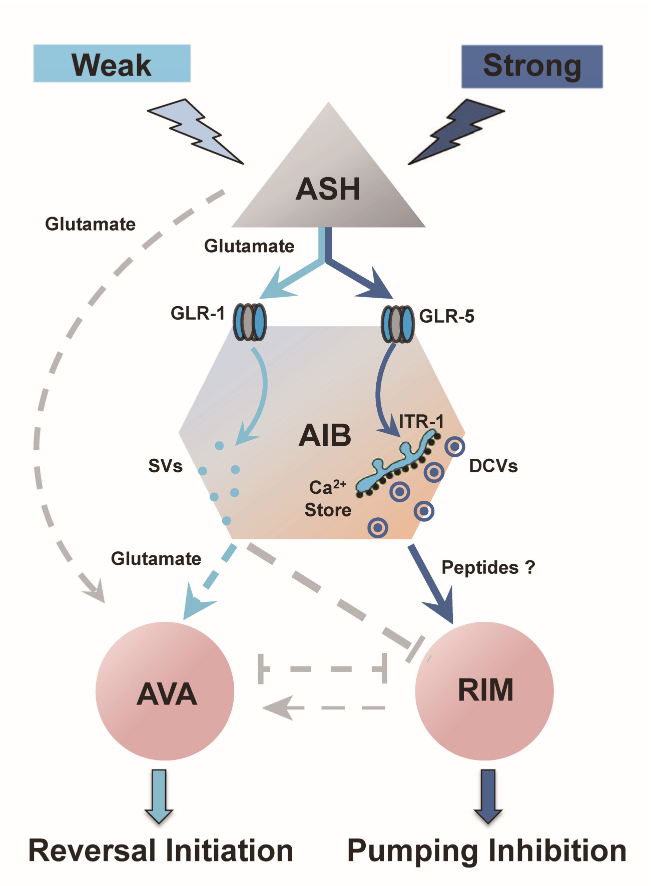

Supplementary Figure 9. A schematic model illustrating how the intensity of quinine inputs were decoded to drive reversal initiation and feeding suppression.

Different concentrations of quinine are encoded by distinct $[Ca^{2+}]_i$ patterns in the same primary neuron (ASH) and downstream interneuron (AIB). Increasing concentration of quinine causes gradual activation of the ASH neurons and gradually increased glutamate release. Post-synaptically, different amount of glutamate release is sensed by two glutamate receptors with different activation thresholds and kinetics: lower amount of glutamate release (i.e., 1 mM quinine input) is sensed by the low-activation threshold, fast-inactivation GLR-1 receptor, while higher amount of glutamate release (i.e., 4-5 mM quinine input) is sensed by the high-activation threshold, slow-inactivation GLR-5 receptor. The activation of these two glutamate receptors in turn triggers differential behavioral outputs: reversal initiation and pumping inhibition. GLR-1 induces small, transient $[Ca^{2+}]_i$ elevations in the AIB neurons, which is likely mediated by Ca^{2+} influx through GLR-1 channels, and

triggers glutamate release via synaptic vesicles (SVs), thereby promoting reversal. In contrast, GLR-5 induces large, sustained $[Ca^{2+}]_i$ elevations in the AIB neurons, which is mediated by an unconventional metabotropic mechanism involving G proteins and Ca^{2+} release from IP3-sensitive Ca^{2+} stores, thus in turn triggers release of neuropeptides from dense core vesicles (DCVs), activates the downstream interneuron RIM and results in feeding suppression. Dashed lines represent proposed connections from previous studies[3-5].

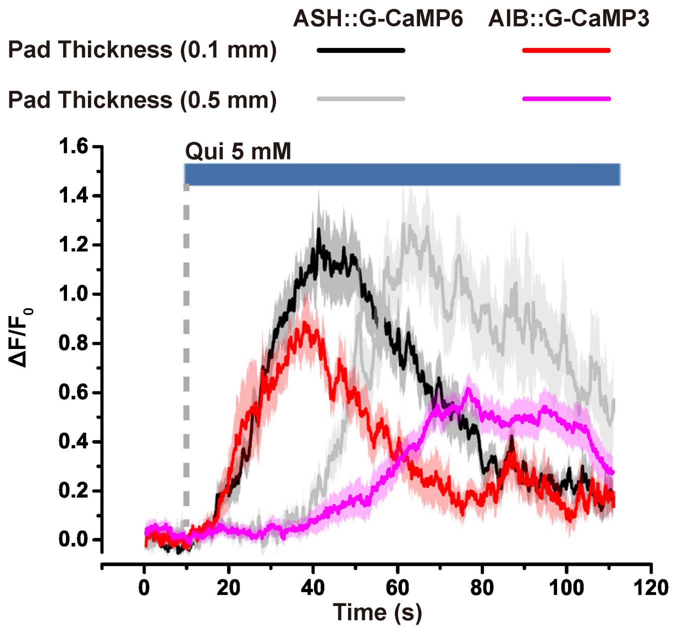

Supplementary Figure 10. Comparison of ASH and AIB $[Ca^{2+}]_i$ responses in agar pads of different thickness.

Averaged $[Ca^{2+}]_i$ responses induced by 5 mM quinine in ASH and AIB neurons of freely moving worms on agar pads of thickness ~0.1 mm and ~0.5 mm, respectively. To estimate the thickness of the agar pads, we pasted layers of sticky tape on the edge of a glass slide, and the thickness of one layer of sticky tape was measured as 0.1 mm. A droplet of liquid agar was added on the glass slide before sandwiching with another glass slide. Worms were crawling between a coverslip and an agar pad, and quinine droplets were added to the other side of agar pad to perfuse through agar and reach the worm. The shades around traces indicate error bars (SEM, $n \geq 7$).

Supplementary References

1 Esposito, G., Di Schiavi, E., Bergamasco, C., and Bazzicalupo, P. Efficient and cell specific knock-down of gene function in targeted C. elegans neurons. *Gene* 395, 170–176 (2007).

2 Hilliard, M. A., Bargmann, C. I. & Bazzicalupo, P. *C. elegans* responds to chemical repellents by integrating sensory inputs from the head and the tail. *Current biology : CB* **12**, 730-734 (2002).

3 Gordus, A., Pokala, N., Levy, S., Flavell, S. W. & Bargmann, C. I. Feedback from network states generates variability in a probabilistic olfactory circuit. *Cell* **161**, 215-227, doi:10.1016/j.cell.2015.02.018 (2015).

4 Guo, Z. V., Hart, A. C. & Ramanathan, S. Optical interrogation of neural circuits in *Caenorhabditis elegans*. *Nature methods* **6**, 891-896, doi:10.1038/nmeth.1397 (2009).

5 Piggott, B. J., Liu, J., Feng, Z., Wescott, S. A. & Xu, X. Z. The neural circuits and synaptic mechanisms underlying motor initiation in *C. elegans*. *Cell* **147**, 922-933, doi:10.1016/j.cell.2011.08.053 (2011).
